# Psychotherapeutic and Psychiatric Intervention in Patients With COVID-19 and Their Relatives: Protocol for the DigiCOVID Trial

**DOI:** 10.2196/39080

**Published:** 2022-11-16

**Authors:** Filippo Cantù, Bruno Biagianti, Ilaria Lisi, Elisa R Zanier, Nicola Bottino, Chiara Fornoni, Francesca Gallo, Valeria Ginex, Valentina Tombola, Silvana Zito, Elisa Colombo, Nino Stocchetti, Paolo Brambilla

**Affiliations:** 1 Department of Pathophysiology and Transplantation University of Milan Milano Italy; 2 Department of Research and Development Posit Science Corporation San Francisco, CA United States; 3 Laboratory of Acute Brain Injury and Therapeutic Strategies Department of Neuroscience Istituto di Ricerche Farmacologiche Mario Negri Istituto di Ricovero e Cura a Carattere Scientifico Milano Italy; 4 Department of Anaesthesia and Critical Care Fondazione Istituto di Ricovero e Cura a Carattere Scientifico Ca' Granda Ospedale Maggiore Policlinico Milano Italy; 5 Department of Neurosciences and Mental Health Fondazione Istituto di Ricovero e Cura a Carattere Scientifico Ca' Granda Ospedale Maggiore Policlinico Milano Italy; 6 Respiratory Unit and Cystic Fibrosis Adult Center Fondazione Istituto di Ricovero e Cura a Carattere Scientifico Ca’ Granda Ospedale Maggiore Policlinico Milano Italy

**Keywords:** telepsychiatry, telemedicine, COVID-19, mental health, digital mental health, digital support, clinical outcome, telehealth, psychiatric health, health intervention

## Abstract

**Background:**

The COVID-19 pandemic is negatively impacting the mental health of both patients with COVID-19 and the general population. As current guidelines are limiting in-person contacts to reduce the spread of the virus, the development of a digital approach to implement in psychiatric and psychological consultations is needed. In this paper, we present the DigiCOVID protocol, a digital approach to offer remote, personalized psychological and psychiatric support to former or current patients with COVID-19 and their relatives.

**Objective:**

The main goal of this project is to evaluate the feasibility, acceptability, and usability of the DigiCOVID protocol. Furthermore, we also aim to assess the impact of the abovementioned protocol by means of pre-post changes in psychological clinical variables.

**Methods:**

Participants undergo an initial telephonic screening to ensure inclusion criteria are met. Secondly, participants complete a video-assisted neuropsychological IQ test as well as web-based self-reports of health and general well-being. Participants are then assigned to a psychotherapist who offers 8 teletherapy sessions. At the end of the therapy cycle, the web-based questionnaires are administered for a posttreatment evaluation.

**Results:**

As of April 2022, we enrolled a total of 122 participants, of which 94 have completed neuropsychological tests and web-based questionnaires.

**Conclusions:**

Our study aims at testing the feasibility and preliminary efficacy of DigiCOVID, a remote telemedicine protocol for the improvement of psychological and psychiatric health in patients with COVID-19 and their relatives. To date, the approach used seems to be feasible and highly customizable to patients’ needs, and therefore, the DigiCOVID protocol might pave the way for future telepsychiatry-based interventions.

**Trial Registration:**

ClinicalTrials.gov NCT05231018; https://clinicaltrials.gov/ct2/show/NCT05231018?term=NCT05231018 &draw=2&rank=1

**International Registered Report Identifier (IRRID):**

DERR1-10.2196/39080

## Introduction

The COVID-19 pandemic caused by SARS-CoV-2 virus is an unprecedented event both for the general population and health care workers.

Northern Italy has rapidly emerged as one of the epicenters of the first COVID-19 outbreak. The hospital in which our trial was designed and implemented, the Fondazione Istituto di Ricerca e Cura a Carattere Scientifico (IRCCS) Ca’ Granda Ospedale Maggiore Policlinico in Milan, was completely reorganized to face new cases in the intensive care unit, paralleled by a large-scale optimization of the entire health care system [1,2].

Current literature is focusing on the connections between COVID-19 and psychiatry, in particular concerning the development of posttraumatic stress disorder and related conditions in patients who contracted the disease [3]. Furthermore, evidence suggests that the pandemic itself might be a potential trigger for various other mental health diseases in the general population, with higher rates of psychological deterioration [4], a probable impact on the development of first episodes of psychosis [5], obsessive compulsive disorder [6], depression [7], anxiety [8], phobic behaviors [9], addictions [10], and general health care workers distress [11,12].

In such a historical moment, it appears of paramount importance to prevent rather than treat psychological distress with every possible means available despite the limitations associated with in-person visits, social distancing, and isolation [13,14]. In fact, current ‘shelter-in-place’ guidelines and restrictions implemented by the public health system to limit the diffusion of the virus are significantly reducing in-person psychological and psychiatric consultations [15]. On the 24th of March 2020, the Ministry for Technological innovation and Digitalization, the Ministry of Health, the National Institutes of Health, and the World Health Organization together published a formal invitation to use telemedicine technologies more thoroughly in clinical practice [16].

A multitude of digital approaches have been tentatively operationalized worldwide to meet clinical needs, with COVID-19 serving as a catalyst for a change in the way instruments are used daily in health care settings [17]. Telepsychiatry, an innovative approach that consists of remote consultations and evaluations for mental health symptoms, has become a reliable alternative to face-to-face assessments, thus adapting rather than succumbing to COVID-19 [18,19], and assisting those affected by the psychosocial consequences of the pandemic [20].

Aside from patients with COVID-19, the psychological aftermath of this pandemic to a similar degree interests those who have not contracted the virus but have clinical conditions to manage, have lost family members or peers, are unable to enjoy the physical presence of their families, and have been living in restricted conditions for months. This phenomenon is likely due to the general psychological impact of the pandemic event per se and the systemic modifications in family relationships, with high amounts of stress also in healthy relatives [21,22]. Incidence and prevalence of psychological distress have been skyrocketing beyond the possibilities of any mental health system to intervene with in-person services. This segment of the population has profound unmet clinical needs and increasingly requests access to psychiatric services.

In this paper, we aim to describe DigiCOVID, a digital mental health protocol designed to offer remote, personalized support to former or current patients with COVID-19 and their relatives. DigiCOVID includes psychotherapy sessions and psychiatric consultations depending on participants’ needs. An observational longitudinal study in patients with COVID-19 and their relatives is being conducted to investigate whether the intervention described above is feasible, usable, and acceptable for the target population. Outcome measures include engagement rates with psychotherapy sessions and psychiatric consultations, overall program completion rates, reported adverse effects, usability ratings, and clinician burden. The secondary objectives aim to (1) evaluate whether DigiCOVID induces changes in self-reports of anxiety, depression, insomnia, trauma, and quality of life and (2) determine whether DigiCOVID impacts patients with COVID-19 and their family members differently.

## Methods

### Overall Design and Timeline

The study is conducted jointly by the units of pneumology, internal medicine, psychiatry, and the laboratory of brain injury and therapeutic strategies of the Istituto di Ricerche Farmacologiche Mario Negri. Before accessing DigiCOVID, participants are taken through the following steps: first, participants undergo an initial screening to ensure the main inclusion criteria are met. Then, 2 trained neuropsychologists remotely perform neuropsychological tests on patients via Zoom. The tests are aimed at assessing patients’ IQ through the Test di Intelligenza Breve (Italian for brief intelligence test) [23] and standard progressive Raven matrices [24]. Suicidality is assessed via the Columbia Suicide Severity Rating Scale [25]. Finally, participants complete a battery of web-based self-reports that include the following: the 12-item General Health Questionnaire [26], the Impact of Event Scale–Revised [27], the 7-item General Anxiety Disorder assessment [28], the Insomnia Severity Index [29], and the 9-item Patient Health Questionnaire [30]. After completing these assessments, participants are assigned to a trained psychotherapist who offers 8 teletherapy sessions carried out through Zoom.

This model is designed to be applied to patients with COVID-19 during hospitalization, after discharge, as well as during the remission and recovery phases. Similarly, this model is intended to be delivered to people who are dealing with the hospitalization or discharge after COVID-19 of a family member or have lost a family member due to COVID-19. A total of 8 remote, 50-minute, individual psychological sessions are offered weekly using secure videoconferencing software. The severity of the clinical conditions of patients with COVID-19 has largely influenced the sequencing of the intervention.

During all phases of the clinical work, suffering is contextualized both in the light of the recent traumatic experience (eg, bereavement, hospitalization in intensive care, and fear for one’s life or that of a relative) and in the light of historical ways of suffering, so that the patient is able to recognize the meaning of the symptoms experienced.

We considered it appropriate to circumscribe the exploration of different psychological targets within each session, given the unpredictable nature of the course of illness and the possible onset of events that radically change the psychological state of patients and family members. What follows is a very schematic summary that refers to the ‘ideal’ situation, where clinical conditions (of the patient or of the relative of the patient) evolve linearly toward recovery.

Session 1 includes introductions, the exploration of the patient’s current experience, identification of the areas of suffering, and a brief recapitulation of the patient’s pre–COVID-19 psychological functioning. Session 2 attempts to define shared goals for the therapeutic process and creates an initial diagnostic framework to identify unprocessed or unregulated emotions. Session 3 aims to validate the intrapsychic and interpersonal resources associated with a greater degree of adaptation to the stressful situation, including a flexible personality; positive beliefs about the self; identity roles, acceptance, and commitment skills; work functioning; and a solid network of friends, family, or loved ones. In sessions 4 through 6, areas of clinical concern are addressed, and defense mechanisms are investigated. Session 7 aims to integrate the lived experience in the cohesive narrative of the self. In session 8, internal working models or relational patterns that have emerged during therapy closure are discussed, and psychoeducation on relapse prevention is offered. At the end of the abovementioned cycle, participants repeat the battery of web-based self-reports.

This paper follows the SPIRIT guidelines for the trial’s publication, as suggested by the EQUATOR Network. The trial is registered on ClinicalTrials.gov (NCT05231018). Of note, the protocol has been registered as it was designed at the beginning of the project, that is, it did not include relatives but only patients. Given the high number of requests, and because the intervention is customizable to each patient’s clinical needs, we have decided to extend our intervention to relatives, thus explaining the differences between the registered protocol and this methodological paper. Regarding the gap between the registration and the trial start, the internal organization of our research unit does not require the registration of trials that do not involve drugs and placebos. Therefore, the trial has been registered on ClinicalTrials.gov to improve the recruitment process.

### Study Population

Our population of interest includes patients with COVID-19, either previously or currently hospitalized at the Foundation IRCCS Ca’ Granda Ospedale Maggiore Policlinico, and their relatives, according to our inclusion and exclusion criteria.

#### Inclusion Criteria

The inclusion criteria for our study are as follows: (1) age 18-80 years; (2) a positive COVID-19 test at the moment of enrolment for participants in the ‘patients’ group; (3) adequate sensory and motor abilities, without impairments in vision and hearing or handling devices; (4) access to wireless internet technologies; and (5) a good level of Italian in terms of speaking, reading, and writing.

#### Exclusion Criteria

The exclusion criteria for this study are the following: (1) present or past medical history of schizophrenia, schizoaffective disorder, delusional disorder, bipolar disorder, or current substance abuse, all according to the Diagnostic and Statistical Manual Fifth Edition [31]; (2) a diagnosis of cognitive impairment or dementia (eg, mild cognitive impairment, Alzheimer disease, or Parkinson disease); (3) intellectual disability defined by a total IQ <70 as obtained by the Test di Intelligenza Breve [23] or the standard progressive Raven matrices [24]; (4) severe present medical conditions that could interfere with participation; (5) present or past suicidal ideation or commitment; (6) significant impairment in the use of digital and technological devices, questionnaires and test completions, as well as comprehension, or lack of a compliant behavior in the earliest evaluations; and (7) being enrolled in other clinical trials assessing any psychological or experimental pharmacological treatment.

#### Recruitment

As of April 2022, 122 participants have been referred to the study. Participation is voluntary, and an extended informed consent form is signed before any evaluation, assessment, or voice and video call. Consent forms are collected remotely for those who have been discharged and are currently in remission and in person for those hospitalized in a COVID-19 ward of either pneumology, internal medicine, or infectious disease departments.

### Primary and Secondary Outcome Measures

#### Primary Outcome Measures

Efforts will be made to assess all participants who have completed the minimum required intervention activities. For DigiCOVID, the minimum required intervention activities include attending psychotherapy sessions at least 4 times. As the main goal of this project is to evaluate the feasibility, acceptability, and usability of DigiCOVID, we will conduct an analysis of the following primary outcome measures in all intention-to-treat (ITT) participants:

- Assessment completion rate—based on our previous studies, we expect ≥80% of participants will complete the battery of web-based self-reports.
- Usability ratings obtained post DigiCOVID via a 7-point Likert scale questionnaire (ie, mean rating of all responses)—this is a brief and embedded poststudy questionnaire on program satisfaction, clarity, and perceived benefits. Participants will rate each sentence on the following 7-point Likert scale: 1=completely agree; 2=mostly agree; 3=somewhat agree; 4=undecided; 5=somewhat disagree; 6=mostly disagree; and 7=completely disagree. Based on our previous studies, we hypothesize exit survey ratings of at least ≥4.5 (SD 1.5) on the 7-point Likert scale items.
- Reported side effects (ie, raw score)—based on our previous findings, we expect 0 adverse events due to program use.
- Overall program completion rate—based on previous findings, we hypothesize full program completion in ≥70% study participants.

#### Secondary Outcome Measures

The secondary outcome measures will be collected at baseline and immediately after the treatment for all participants. We designed DigiCOVID to improve mental well-being. Therefore, we will measure the impact of the intervention by looking at pre-post changes in the following outcome measures: the 12-item General Health Questionnaire [26] , the Impact of Event Scale–Revised [27], the 7-item General Anxiety Disorder scale [28], the Insomnia Severity Index [29], and the 9-item Patient Health Questionnaire [30]. We expected to observe a significant improvement across all these secondary outcome measures *P*< in both patients with COVID-19 and their family members. To verify these experimental hypotheses, we will conduct the analysis based on the preintervention (baseline) and postintervention data, using parametric and nonparametric statistical tests. The criterion for statistical significance is *P*<.05. Results with *P*<.1 will be described as trends.

### Data Collection

The sources of the research material will consist of data collected through assessment visits and the DigiCOVID app, strictly for research purposes. Participants will be carefully screened for contraindications prior to participation. All intervention data are coded so as not to identify any given participant and be securely stored. Hard copy data from the neuropsychological tests will be stored in a locked file cabinet in a safe location with limited access by authorized personnel or on password-protected computers in locked offices. All data from this trial will be recorded on a secure, web-based software application designed to support data capture for research studies. To achieve robust and unbiased results, data will undergo a rigorous quality control process to ensure consistency in scoring, coding, and accuracy of data entry. Standard data quality procedures will be used, including double scoring, random spot checking of assessments, and electronic data capture. Additionally, 20% of all data folders will undergo a random audit every 3 months. The database will be designed to not allow illegal values in entry. Any outliers will be double-checked with the raw data for accuracy.

### Data Analysis

There are 3 a priori defined analysis populations, including a primary analysis population, a secondary analysis population designed to compare effect sizes in populations with no missing data, and a population who completed all study visits. The 3 populations are as follows:

- ITT population: this is the a priori primary analysis population, defined as including all participants who attended at least 4 remote psychotherapy sessions.
- Intention-to-treat fully evaluable (ITT-FE) population: this is a secondary analysis population, defined as including all members of the ITT population who complete a postintervention visit. Note that a participant may complete a specific visit but have missing data for a test, in which case the participant is in the overall ITT-FE population but does not contribute data to the ITT-FE population for that visit (eg, the number of evaluable cases for a specific test on a specific visit may be smaller than the ITT-FE population for that visit because of missing data).
- Intention-to-treat completers (ITT-C) population: this is a secondary analysis population, defined as including all members of the ITT-FE population who complete all intervention sessions. Note that the ITT-C population is a strict subset of the ITT-FE population; a person who completes the treatment but does not complete the postintervention evaluation visit is not a member of the ITT-C population.

We also plan to perform different analyses in order to better describe the demographics of patients and relatives and to test whether there are significant differences in terms of response to our psychotherapy intervention.

### Ethics Approval

This study was approved by our local Ethics Committee (IRCCS Ca’ Granda Ospedale Maggiore Policlinico) on October 28, 2020 (protocol code 962_2020, 03/11/2020).

## Results

This project has been funded by Fondazione Cariplo under Award number 2020-1366, in June 2020. As of April 2022, we enrolled a total of 122 subjects, of which 94 have completed neuropsychological tests and web-based questionnaires; data analyses are currently completed in terms of preliminary results, and we expect results to be published by the end of 2022.

## Discussion

### Expected Outcomes

This paper describes the methodology adopted to remotely assess and promptly treat psychiatric symptoms in a sample of patients with COVID-19 and their first-degree relatives. This digital approach is showing its innovation potential by helping to manage the psychological burden caused by the pandemic in patients with COVID-19 and their relatives.

We expect, as a prediction of our hypotheses, that the DigiCOVID protocol will result in a feasible approach; such a claim is clearly a hopeful statement, yet it is based on the quick, efficient, and technological step procedure described earlier. In terms of descriptive statistics, we might observe slight differences in terms of symptoms between patients and relatives (ie, in the posttraumatic scale, which we expect to be higher in patients than in relatives). In conclusion, we also expect the DigiCOVID protocol to be effective for both patients and their relatives in diminishing psychological distress. Future directions might involve the standardization of the DigiCOVID protocol in COVID-19 wards, as part of a complete program for the treatment of patients with COVID-19 and participants who might not have the opportunity to move to see a professional if remote visits are not possible.

To date, we are completing the recruitment, and we are expecting future and conclusive analyses to determine the efficacy and effectiveness of the protocol.

### Strengths

This project has several lines of innovation. First, the length of the intervention is in line with services routinely offered by the Italian National Health Service (8-week cycle of psychotherapy sessions), which makes the implementation of this remote approach feasible and acceptable. Second, thanks to the data from the self-reports collected before the intervention start, psychotherapists have the opportunity to rapidly customize treatment goals and spare time usually spent collecting past and current psychiatric history. Third, the remote psychological support offered to first-degree relatives can increase personal resources, positively impact the resources of the family, and reinforce familiar bonds, ultimately producing better psychological prognoses for patients. If proven successful and efficacious, this intervention protocol could be standardized and disseminated at a large scale, helping clinicians remotely treat psychological distress, thereby alleviating at large the mental health consequences of the COVID-19 pandemic.

### Limitations

Some limitations have emerged as we operationalized this project. First, we observed a lack of motivation in some patients, likely caused by the remote technology-based approach. Second, even if telepsychiatry has been proven efficacious in assessing patients remotely, psychologists and psychiatrists are asked to adapt their methods of assessment, diagnosis, and treatment to new means of communication. The dissemination of this digital approach at a national level may require additional training for mental health professionals to ensure impact and effectiveness of this promising intervention.

## Abbreviations

IRCCS: Istituto di Ricercar e Cura a Carattere Scientifico
ITT-C: intention-to-treat completers
ITT-FE: intention-to-treat fully evaluable
ITT: intention-to-treat

## Data Availability

The data sets generated and analyzed during this study are available from the corresponding author upon reasonable request.

## References

[1] Grasselli G, Pesenti A, Cecconi M (2020). Critical care utilization for the COVID-19 outbreak in Lombardy, Italy: early experience and forecast during an emergency response. JAMA.

[2] Madonna D, Enrico P, Ciappolino V, Boscutti A, Colombo E, Turtulici N, Cantù F, Cereda G, Delvecchio G, De Falco S, Chierichetti M, Savioli M, Grasselli G, Brambilla P (2022). Factors associated with severity of delirium complicating COVID-19 in intensive care units. Front Neurol.

[3] Zhu Z, Xu S, Wang H, Liu Z, Wu J, Li G COVID-19 in Wuhan: immediate psychological impact on 5062 health workers. medRxiv.

[4] Fusar-Poli P, Brambilla P, Solmi M (2020). Learning from COVID-19 pandemic in Northen Italy: impact on mental health and clinical care. J Affect Disord.

[5] Esposito C, D'Agostino A, Dell Osso B, Fiorentini A, Prunas C, Callari A, Oldani L, Fontana E, Gargano G, Viscardi B, Giordano B, D'Angelo S, Wiedenmann F, Macellaro M, Giorgetti F, Turtulici N, Gambini O, Brambilla P (2021). Impact of the first Covid-19 pandemic wave on first episode psychosis in Milan, Italy. Psychiatry Res.

[6] Davide P, Andrea P, Martina O, Andrea E, Davide D, Mario A (2020). The impact of the COVID-19 pandemic on patients with OCD: effects of contamination symptoms and remission state before the quarantine in a preliminary naturalistic study. Psychiatry Res.

[7] Fitzpatrick KM, Harris C, Drawve G (2020). Living in the midst of fear: depressive symptomatology among US adults during the COVID-19 pandemic. Depress Anxiety.

[8] Ozamiz-Etxebarria N, Dosil-Santamaria M, Picaza-Gorrochategui M, Idoiaga-Mondragon N (2020). Niveles de estrés, ansiedad y depresión en la primera fase del brote del COVID-19 en una muestra recogida en el norte de España. Cad Saúde Pública.

[9] Asmundson GJ, Taylor S (2020). Coronaphobia: fear and the 2019-nCoV outbreak. J Anxiety Disord.

[10] Panchal N, Kamal R, Orgera K, Cox C, Garfield R, Hamel L (2021). The Implications of COVID-19 for Mental Health Substance Use.

[11] Fattori A, Cantù F, Comotti A, Tombola V, Colombo E, Nava C, Bordini L, Riboldi L, Bonzini M, Brambilla P (2021). Hospital workers mental health during the COVID-19 pandemic: methods of data collection and characteristics of study sample in a university hospital in Milan (Italy). BMC Med Res Methodol.

[12] Bonzini M, Comotti A, Fattori A, Cantù F, Colombo E, Tombola V, Myslymi E, Gatti M, Stucchi G, Nava C, Bordini L, Riboldi L, Brambilla P (2022). One year facing COVID. Systematic evaluation of risk factors associated with mental distress among hospital workers in Italy. Front Psychiatry.

[13] Galea S, Merchant RM, Lurie N (2020). The mental health consequences of COVID-19 and physical distancing: the need for prevention and early intervention. JAMA Intern Med.

[14] Badawy SM, Radovic A (2020). Digital approaches to remote pediatric health care delivery during the COVID-19 pandemic: existing evidence and a call for further research. JMIR Pediatr Parent.

[15] Orben A, Tomova L, Blakemore S (2020). The effects of social deprivation on adolescent development and mental health. Lancet Child Adolesc Health.

[16] de Girolamo G, Cerveri G, Clerici M, Monzani E, Spinogatti F, Starace F, Tura G, Vita A (2020). Mental health in the coronavirus disease 2019 emergency-the Italian response. JAMA Psychiatry.

[17] Taylor CB, Fitzsimmons-Craft EE, Graham AK (2020). Digital technology can revolutionize mental health services delivery: the COVID-19 crisis as a catalyst for change. Int J Eat Disord.

[18] Smith K, Ostinelli E, Macdonald O, Cipriani A (2020). COVID-19 and telepsychiatry: development of evidence-based guidance for clinicians. JMIR Ment Health.

[19] Balcombe L, De Leo D (2020). An integrated blueprint for digital mental health services amidst COVID-19. JMIR Ment Health.

[20] Chang BP, Kessler RC, Pincus HA, Nock MK (2020). Digital approaches for mental health in the age of covid-19. BMJ.

[21] Ye J (2020). Pediatric mental and behavioral health in the period of quarantine and social distancing with COVID-19. JMIR Pediatr Parent.

[22] Liu CH, Doan SN (2020). Psychosocial stress contagion in children and Families during the COVID-19 pandemic. Clin Pediatr (Phila).

[23] Sartori G, Colombo L, Vallar G, Ruscoli M (1997). Test di Intelligenza Breve per la valutazione del quoziente intellettivo attuale e pre-morboso. La Professione di Psicologo.

[24] Raven J, Raven J (2003). Raven progressive matrices. Handbook of Nonverbal Assessment.

[25] Posner K, Brown GK, Stanley B, Brent DA, Yershova KV, Oquendo MA, Currier GW, Melvin GA, Greenhill L, Shen S, Mann JJ (2011). The Columbia-Suicide Severity Rating Scale: initial validity and internal consistency findings from three multisite studies with adolescents and adults. Am J Psychiatry.

[26] Goldberg DP, Williams P (1988). A User's Guide to the General Health Questionnaire.

[27] Weiss D, Marmar C (1997). The Impact of Event Scale–Revised. APA PsycNet.

[28] Spitzer RL, Kroenke K, Williams JBW, Löwe B (2006). A brief measure for assessing generalized anxiety disorder: the GAD-7. Arch Intern Med.

[29] Morin C, Belleville G, Bélanger L, Ivers H (2011). The Insomnia Severity Index: psychometric indicators to detect insomnia cases and evaluate treatment response. Sleep.

[30] Kroenke K, Spitzer RL, Williams JBW (2001). The PHQ-9: validity of a brief depression severity measure. J Gen Intern Med.

[31] (2013). Diagnostic and Statistical Manual of Mental Disorders: Fifth Edition DSM-5.

